# Toxicological assessments of agrochemical effects on stingless bees (Apidae, Meliponini)

**DOI:** 10.1016/j.mex.2020.100906

**Published:** 2020-04-30

**Authors:** Lorena L. Botina, Rodrigo C. Bernardes, Wagner F. Barbosa, Maria Augusta P Lima, Raul Narciso C. Guedes, Gustavo F. Martins

**Affiliations:** a Departamento de Entomologia, Universidade Federal de Viçosa, Viçosa, MG 36570-900, Brazil; b Departamento de Biologia Animal, Universidade Federal de Viçosa, Viçosa, MG 36570-900, Brazil; c Departamento de Biologia Geral, Universidade Federal de Viçosa, Viçosa, MG 36570-900, Brazil

**Keywords:** Ecotoxicology, *In vitro* rearing, Meta-analysis, Survival analysis, Wild bees

## Abstract

Bee pollination is crucial for ecosystem maintenance and crop production. The ubiquity of bee pollinators in agricultural landscapes frequently results in their exposure to agrochemicals, which has been associated with their decline. Stingless bees are wild pollinators restricted to the Pantropical region, and like honey bees, are suffering colony losses. However, stingless bees and honey bees do not show the same behaviors, therefore, methods used for risk assessment of honey bees cannot be utilized on stingless bees. Herein, we describe protocols to standardize methods that allow for the exploration of lethal and sublethal effects of agrochemicals via acute and chronic exposure of stingless bees. The *in vitro* rearing used for chronic exposure from the egg to the adult stage proved to be effective in obtaining relevant screenings. In addition, we performed a meta‐analysis and summarized the results of toxicological studies conducted with the protocols described. The meta-analyses indicated a reduction in survival under acute and chronic exposures to agrochemicals, and revealed that our protocols for toxicological assessments did not have publication bias for either acute or chronic exposure. These findings proved that these standardized protocols are reliable for toxicological research on stingless bee.

## Graphical abstract

**Figure:**
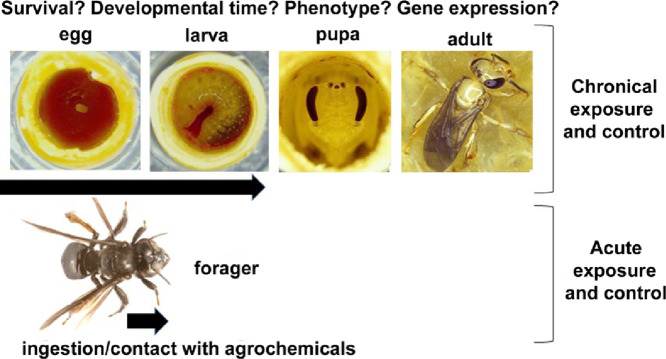

Specifications Table

**Table:** 

Subject Area	Environmental Science
More specific subject area	Ecotoxicology
Protocol name	Exposure of stingless bees to agrochemicals *in vitro*
Reagents/tools	Material/ Equipment **Acute exposure** Incubator (Biochemical oxygen demand) – Ethiktechonology Analytical precision scale – Shimadzu Thermohygrometer – Incoterm Micropipette (200 µL and 1000 µL) – HTL Plastic containers Microtubes 2 mL Glass beakers of several sizes Sucrose Filter paper – J. Prolab Paper towel Glue stick Sandpaper Nitrile gloves – Kevenoll Pipette tips (200 µL and 1000 µL) – Kasvi Glass jars or Erlenmeyer Distilled water Agrochemicals (see description in Additional information) **Chronic exposure** Incubator (Biochemical oxygen demand) – Ethiktechonology Analytical precision scale – Shimadzu Thermohygrometer – Incoterm Surgical aspirator – Aspiramax Multiwell Cell Culture Plates (48 and 96 wells) – Kasvi Handsaw Forceps and micro-dissection forceps Glass chambers or desiccator Repeating pipette and tips (1250 µL) – Gilson UV light Glass beakers of several sizes Nitrile gloves – Kevenoll Face mask Laminar flow hood Petri dish Pipette tips – Kasvi Micropipette (200 µL and 1000 µL) – HTLN ylon Microtubes 2 mL Nontoxic paint – Brasilux Cotton Plastic containers Lamp Ethanol (70% v/v) Honey bee wax NaCl Distilled water Bleach (10% v/v) Acetone Agrochemicals (see description in Additional information) Wooden tool (Fig. 2)
Experimental design	We performed a meta-analysis based on results from previous published works that used the protocols for ecotoxicological bioassays with the stingless bees. For acute adult exposure, we found five studies with 29 independent controlled bioassays with binary outcomes (mortality) and calculated the risk ratio. For chronic larva exposure, we found six studies with 10 independent controlled bioassays. We measured the effects of chronic exposure on survival and calculated hazard ratios. We measured heterogeneity and, when necessary, subgroup analyses were performed to explain the statistical heterogeneity. The results were validated according to the comparison of the survival rate or mortality rate between bees treated with agrochemical and untreated bees (control) for both acute and chronic exposures.
Trial registration	Not applicable
Ethics	Not applicable
Value of the protocol	• Protocols described serve as a baseline to perform acute and chronic exposure of stingless bees to agrochemicals. • The survival rate of exposed bees varied according to the type of agrochemical, route of exposition, tested species, and developmental stage. • Published papers using our rearing protocol did not have a publication bias for either acute exposure or chronic exposure, indicating the suitability of the protocols.

## General steps

### Required equipment and tools

At the beginning of the experiments, prepare and sterilize the working area and organize all the tools needed for the subsequent bioassays. Clean the working area with 70% ethanol to minimize contamination. All tools and materials should go through the cleaning and sterilization process with 70% ethanol or under UV light before starting the tests. All glassware should be cleaned with acetone before and after use to prevent contamination by agrochemical residues (Table of materials).

### Handling agrochemicals

Agrochemicals need special care and handling. The following practices describe safe, responsible, and effective use and handling procedures. Always read the agrochemical label carefully before using the product and follow label instructions to avoid potential associated hazards. Minimize the risk by using safe working procedures and provide suitable personal protective equipment (nitrile gloves, facemasks, and lab coats) to avoid spilling and poisoning. Prepare and manipulate the solutions in an airflow control area to avoid inhalation. If any exposure occurs, be sure to follow the first aid instructions on the product label carefully [1].

### Determination of sublethal concentration

Serial dilutions of treatment stock solutions (i.e., the concentrated solution of agrochemicals before dilution) are prepared with distilled water for commercial agrochemical formulations to represent the environmental degradation of the active compounds. This also allows the assessment of sublethal effects when no lethal effect is observed.

Procedures for the determination of the LC_50_ are based on serial dilutions of stock solutions. The toxicity test is conducted with at least five concentrations to cover the range for LC_50_ estimates [2]. These concentrations are defined in the bioassays of mortality.

In the next sections, protocols for acute exposure using adult bees and chronic exposure using immature bees will be described.

## Adult toxicity test via acute exposure

This section describes techniques for assessing the toxicity of chemical compounds on adult foragers of stingless bees within a maximum period of 72 h of exposure. The bees are treated in small groups with a minimum of 10 and a maximum of 20 individuals per replicate. Each colony is considered a biological replicate of each treatment. This prevents pseudoreplication because individuals of the same colony do not exhibit independence of errors because of the coexistence of half-sister workers and the shared environment. A minimum of three replicates (i.e., three colonies) per treatment is required.

### Preparation of plastic pots and feeders

The capacity of plastic pots should be suitable for the number and size of bees (i.e., 10 bees should be kept in a 250 mL pot and more than ten bees in a 500 mL pot).

Make small holes of ~1 mm diameter in the lids to allow insects to breathe.

Make a hole (~13 mm diameter) on the lower side of each plastic pot to insert the feeders (2 mL microcentrifuge tubes). The hole is then covered by tape until the feeders are inserted to avoid the escape of bees.

**NOTE**: The feeder must fit into the hole so that there is no space to avoid the escape of bees.

Line the inner bottom of the pot with filter paper and scratch the inner walls of the pot with sandpaper to keep bees from slipping. Identify each plastic pot.

Drill the bottom of the feeders (~1.5 or 2 mm diameter) to allow feeding.

**NOTE:** Plastic pots should be properly discarded after use to avoid contamination. They should be placed in a dumpster lined with a sturdy and properly labeled trash bag.

### Preparation of sucrose solution and agrochemical solutions

Dissolve sucrose in distilled water using a glass beaker and mix it with a magnetic stirrer. The sucrose solution is prepared in a 1:1 (w/w) proportion. Prepare the solution on the same day or the day before the test; in the latter case, maintain at ~4 °C.

Prepare agrochemical solutions using the maximum concentration based on commonly used label rates. The agrochemicals are directly diluted on the aqueous sucrose solution to obtain the solutions to be added in the diet for oral exposure or diluted in distilled water for contact exposure.

**NOTE:** The contaminated diets (i.e., sucrose solution + agrochemical solution) must be homogeneous without apparent signs of precipitation.

After preparation, diets can be stored in a freezer under complete darkness for no more than 2–3 h. However, it is recommended to prepare them during the assembly of the experiment.

### Collection and preparation of bees

**Fig. 1:**
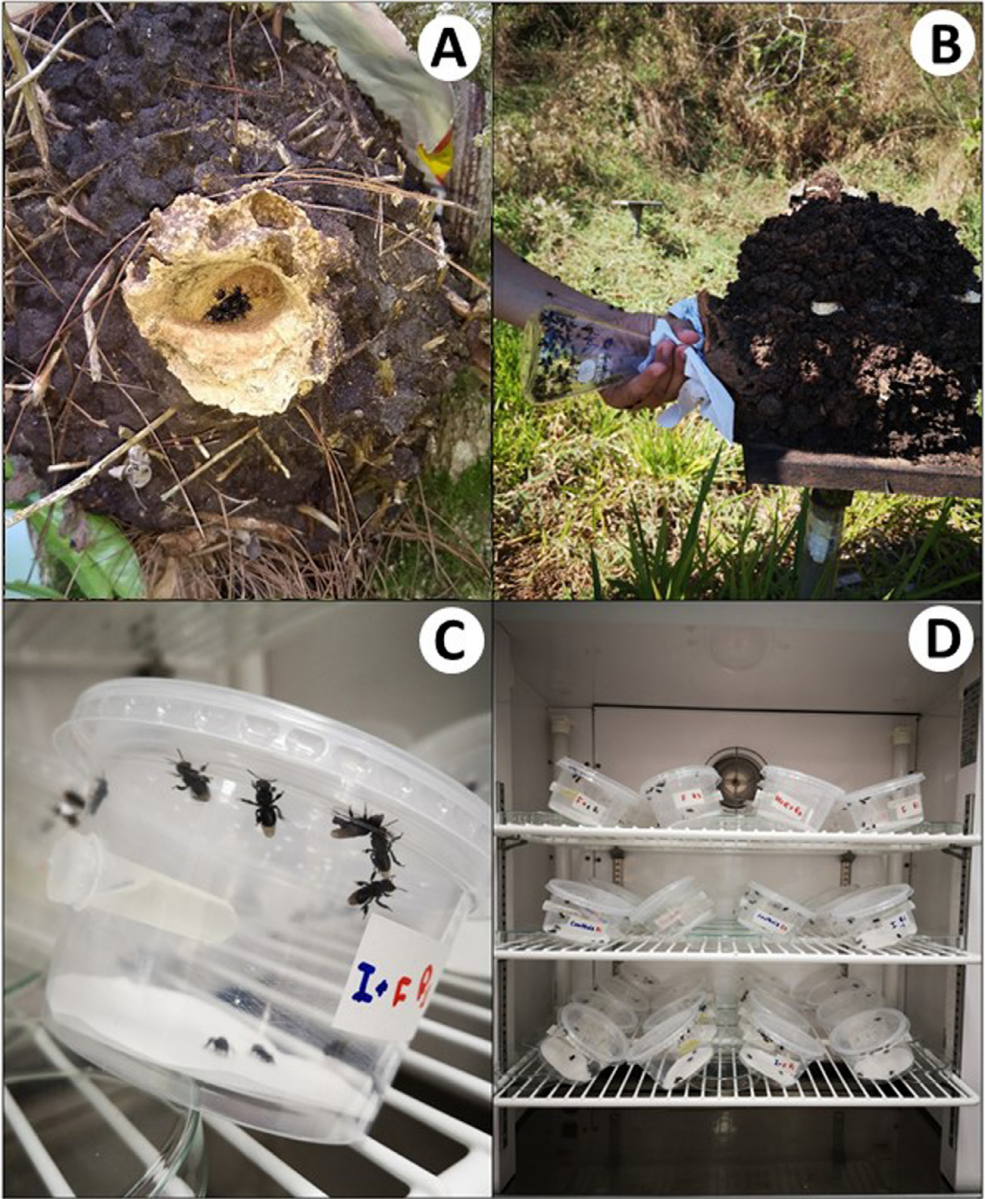
Experimental set-up showing the representation of acute exposure bioassays in stingless bees. (A) a hive of *Partamona helleri*; (B) collection of worker bees at the entrance of the hive; (C) plastic containers with bees; (D) full assembly of the experiment within the incubator.

**NOTE:** Before sampling, lightly tap the bee hive with the hand or a spatula to excite the bees and encourage them to go outside. Use a different jar for each colony to avoid fights/injuries among bees from different colonies.

Collect adult foragers at the hive entrance using glass jars (Fig. 1(A) and (B)).

After catching the required number of bees, quickly close the glass jars with paper wads.

Take the glass jars to the laboratory, anesthetize the foraging bees in the jars with carbon dioxide (CO_2_) and gently transfer them to plastic pots (Fig. 1(C)) previously prepared. The time for anesthetizing should be minimal, no more than 5 s. If you do not have CO_2_, the foraging bees can be anesthetized in a freezer at ~−20 °C for a few minutes (1–2 min) and then transfer to pots. Another way to transfer bees is by using a red-light lamp in a completely dark room. This requires a cage covered with tulle. The bees are released inside the cage and transferred to the pots. The last procedure is recommended for sublethal assessment to minimize handling stress.

Place the pots in an incubator (Fig. 1(D)) in complete darkness under controlled conditions of temperature and humidity, according to the species (Table 1).

**Table 1:** Overall features of four species of stingless bees for maintaining adults in the laboratory.

Species	Ideal colony conditions	Fasting	Survival of controls (%)
*Friesella schrottkyi*[19]	25 ± 2 °C; 70 ± 10% RH	2h	100
*Melipona quadrifasciata*[20]	25 ± 2 °C; 70 ± 10% RH	1h	100
*Partamona helleri*[20], [21], [22], [23]	28 ± 1 °C; 75 ± 5% RH	1h	100
*Scaptotrigona xanthotricha*[22]	25 ± 2 °C; 70 ± 10% RH	1h	100

**Note:** Each pot should contain between 10 and 20 bees. Bees should be obtained from healthy colonies and without previous exposure to agrochemicals. Moribund bees affected by handling should be discarded. A fasting period (Table 1) before the exposure is necessary to acclimatize the bees and encourage them to feed on the contaminated diet.

### Use of agrochemical solutions

Acute exposure to agrochemical solutions can be done through contact or oral exposure.

**Note:** Weigh all feeders at the beginning and end of the exposure on an analytical scale to estimate the average food intake. Plastic pots with feeders but without bees should be maintained under the same experimental conditions to estimate the weight loss of the diet by evaporation, which will be used to correct the rate of food intake per group. The plastic pots should be slightly tilted in an incubator in a way that allows food availability from the feeder.

#### Contact exposure

Impregnate homogeneously and completely the inner walls of plastic pots with the agrochemical solutions using an artist's airbrush coupled with an air pump. Use uncontaminated water as control.

**Note:** The amount of agrochemical solution to impregnate the pots should be adjusted to the volume of pure water necessary to cover the walls without runoff.

Dry the sprayed pots for 2 h in a dark exhausting chamber at 25 ± 3 °C [3].

Supply each group of bees with an uncontaminated diet (i.e., without agrochemical) (1.5 mL), which serves as a food source provided *ad libitum* during the test time using one drilled microtube as a feeder.

Transfer anesthetized bees to agrochemical-impregnated pots. Immediately, add a feeder with a 1.5 mL uncontaminated diet and place them inside the incubator in complete darkness under controlled conditions.

After the exposure time, transfer the treated bees to an untreated pot and supply them with a new and uncontaminated diet.

Transfer the control bees to pots free of agrochemicals.

#### Oral exposure

For oral exposure, the contaminated diet is offered *ad libitum* using drilled 2 mL microtubes as feeders that will be inserted across a hole into the plastic pot for a given time.

**Note:** Exposure time will vary according to the experiment and species, which usually varies between 3 and 24 h. This is related to the fasting period and feeding behavior of each species.

Transfer anesthetized bees into pots in the incubator and fast them for 1–3 h (Table 1) under darkness and adjusted temperature and relative humidity (RH).

Add a feeder with a contaminated diet to each pot, which is removed within 24 h and replaced with an uncontaminated diet until the end of the experiment.

Supply the control group with a diet without an agrochemical. The control provides the evaluation standard in the assessments.

### Mortality assessment and observations

In all treated and control groups, mortality can be recorded at 16 h, 12 h, 24 h, 36 h, 48 h, 60 h, and 72 h. Usually, mortality values used to calculate the LC_50_ or other sublethal doses are recorded up to 24 h.

The treated adult is considered dead if unable to move or stand upright.

Raw data should be summarized in a tabular or figurate form of the survival curves, which show the number of dead bees at each observation time for each treatment.

All abnormal behavioral effects observed during the testing period should be recorded to detect possible sublethal effects.

To validate the test, the average mortality in control groups should not exceed 10–15% at the end of the test. The mortality of the treated group should meet the specified range: almost 25% for the lower concentration up to 80–100% for the higher concentration of the agrochemical. Data from tests failing to meet these standard criteria should not be used and a full study should be conducted exploring other concentrations [2].

## Larval toxicity test via chronic exposure

The *in vitro* rearing of bees allows for toxicity assessment of the exposure to the contaminated diet by larvae from their first larval instar until the end of the larval stage. In nature, the foragers can feed and carry contaminated food (e.g., water, nectar, and pollen) to the hive, which can serve as larval food. In chronic exposure assays, each colony comprises a biological replicate. Tests must include a minimum of three replicates for each treatment. Each replicate should use a minimum of 15 larvae. The following protocol for the rearing of stingless bees depicts adaptations from protocols described elsewhere [4], [5], [6], [7], [8], with the goal of obtaining the highest survival rate for the control groups. This section describes the *in vitro* rearing protocol suitable for three stingless bee species (*Melipona quadrifasciata, Partamona helleri*, and *Trigona spinipes*).

### Prepare a safe and sterile workspace

**NOTE:** In addition to the sterilization described above, the following procedures should be conducted:

Clean the desiccators, incubators, polyethylene microplates (in case it is not a new plate), and forceps with a bleach solution (10% sodium hypochlorite) and pure water. Let them dry completely.

Sterilize all tools, materials, and supplies with 70% ethanol and leave at least 30 min under UV light under a flow hood to minimize possible contamination (Table of materials).

Wash hands thoroughly with soap and water. Wear nitrile gloves and a face mask.

### Prepare multiwell plates

Make artificial brood cells with honey bee wax placed in the wells of polyethylene multiwell cell culture plates. Each larval cell is covered with a wax cap.

**Fig. 2:**
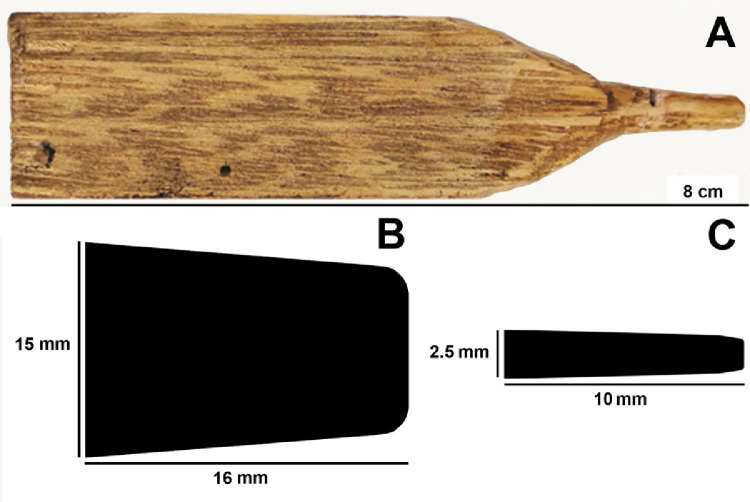
Wooden tool used to shape artificial brood cells of *Partamona helleri* (workers) (A). Schematic representations of cone-shaped tips of the tools used to shape brood cells of workers of *Melipona quadrifasciata* and queens of *P. helleri* (B), and brood cells of workers of*P. helleri* and *Trigona spinipes* (C).

**Note:** Honey bee wax can be obtained from an apiary (preferably located where there is no application of agrochemicals in the neighborhood). The wax is filtered to remove impurities, then heated and shaped using a tool made with a piece of wood with a cone-shaped tip (Fig. 2). The diameter and height of the artificial cells should resemble those in natural comb cells. Thereby, the type of plates should be selected according to the size of the natural comb cell of each species. Usually, the size of the artificial cells is larger than the natural cells. The dimensions of the tip of the wooden tools also depends on the size of comb cell (Table 2). The wax cap is made with a sheet of wax.

**Table 2:** Overall features of three species of stingless bees for rearing in the laboratory.

Species	Ideal temperature	Larval food in brood cell (µL)	Duration to emergence (days)	Survival of controls (%)	Plates	Cone-shaped tip of the wooden tool (diameter × length - mm)
*Melipona quadrifasciata*[5,^7^,17]	28 ± 2 °C	140 (works)	41 ± 1	90	24 wells	14–15 × 16
*Partamona helleri*[6,18]	28 ± 2 °C	40 (works)	46 ± 1	80	96 wells	2–2.5 × 10
		80 (queens)	39 ± 1	85	24 wells	14–15 × 16
*Trigona spinipes*[4]	34 ± 2 °C	36 (works)	34 ± 0.41	90	96 wells	2–2.5 × 10

Place the polyethylene microplate with the artificial cells under a UV light for 30 min before transferring the larval diet.

**Note:** Use separate plates for treatment and control groups to avoid cross contamination.

### Prepare solutions of agrochemicals

Prepare agrochemical solutions using the maximum concentration of each agrochemical that corresponds to the field rates commonly used. The agrochemical formulations are directly diluted in distilled water using glass beakers and stored in a freezer until use.

**Note:** The agrochemical solutions must be homogeneous without apparent signs of precipitation.

### Collection of brood combs

**Fig. 3:**
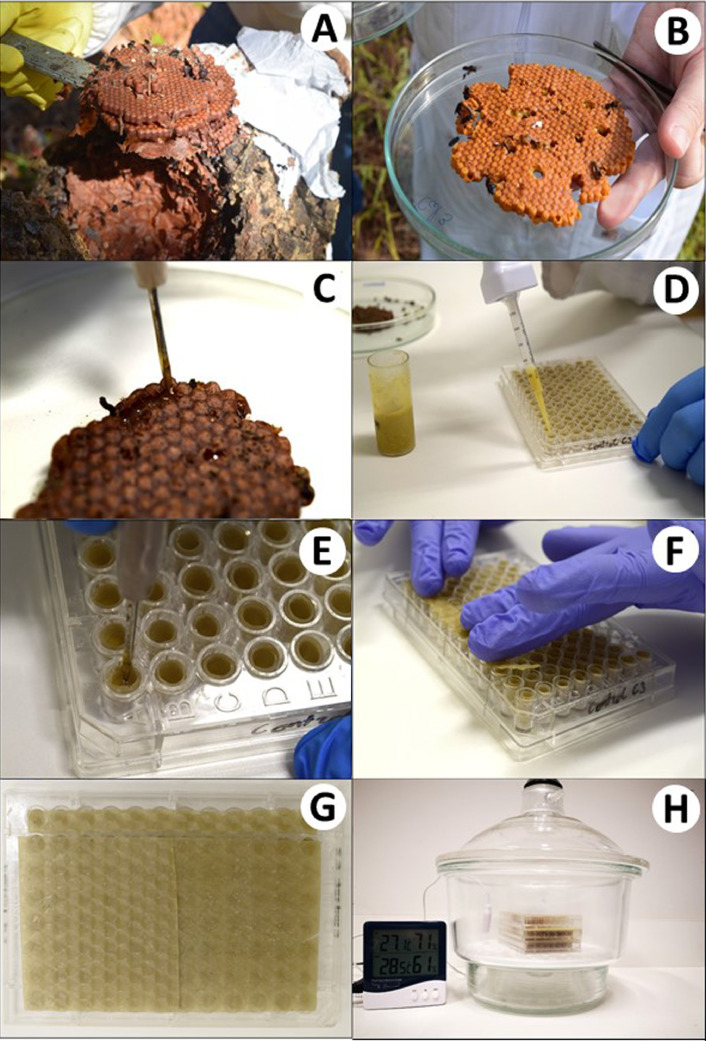
Experimental set-up showing the representation of the chronic larva exposure through *in vitro* rearing of stingless bees. (A) Collection of brood combs from the hive (B) Transport of the brood combs; (C) larval food distribution on microplates; (D) and (E) eggs grafting to pre-filled cell with larval food, (F) and (G) artificial brood cells covered with a wax cap and (F) incubator containing desiccators with microplates. Full assembly of the experiment.

**Note:** For sampling of species with aggressive behavior (e.g., *P. helleri* and *T. spinipes*), wear effective protective clothing, such as a beekeeper jumpsuit.

Collect brood combs of *M. quadrifasciata* directly from rational box hives (Fig. 3(A) and (B)). In the case of *P. helleri* and *T. spinipes*, cut the hive in the middle using a handsaw. The hive should be handled carefully to avoid sudden movements that could drop the eggs.

Using a spatula or nylon tool, gently remove the combs and transfer them into a clean Petri dish on a plastic tray (Fig. 3(B)).

Reinstall the cover of the hive and place it back in the original order and orientation.

Carefully and rapidly transport the combs to the lab under controlled conditions of temperature and RH similar to those of the hive. Never shake the combs while manipulating or transporting to avoid tipping eggs.

### Collection of larval food and preparation of contaminated larval diet

Carefully open the cap of brood cells starting from the center to the outside of the combs using micro-dissection forceps. Oviposition occurs from the center to the edge of the combs; thus, in large combs, larvae tend to be found in the center of the combs and eggs at the periphery.

**Note:** The room should be at ~70% RH and 25 °C or more to avoid food dehydration and death of the eggs.

Discard the larvae and proceed with diet collection using a surgical aspirator (Fig. 3(C)). Transfer the food (or larval diet) to a sterilized and labeled glass vessel.

**Note:** Larval diet should be collected from brood cells with eggs or first instar larvae. The diet of cells containing advanced instar larvae is more dense and young larvae (i.e., used in the assays) cannot feed.

Homogenize the larval diet by making gentle circular movements in the glass vessel.

In separate glass beakers, add agrochemical solution directly into the larval diet. The control treatment is prepared with distilled water (solvent) only.

**Note:** The dilution of the agrochemical solution in the larval food must not exceed 10% of the final volume. It is also necessary to use a constant solution volume for all treatments to have a constant ratio between the larval food and agrochemical solution [9].

Deposit the contaminated larval diet at the bottom of each artificial cell (Fig. 3(D)). Provide the same amount of diet that larvae receive under natural conditions in the colony to allow their complete development. The larval diets should be released slowly from the repeating pipette to avoid bubbles. The amount of larval diet required for each species is shown in Table 2.

### Egg grafting and rearing bees

Gently remove the eggs from uncovered brood cells and place them vertically on the larval diet. A cold light source can be used to facilitate viewing of the eggs in the brood cells. The forceps are cleaned with a paper towel for each grafting to avoid the tipping of the eggs.

**Note:** Eggs tipped or grafted improperly should be discarded and replaced with new ones. Each artificial brood cell receives only one egg (Fig. 3(E)).

Cover the artificial brood cell with a wax cap and transfer the rearing microplates into a desiccator provisioned with Petri dishes (Fig. 3(F)–(H)) containing sterilized water to maintain RH at ~95%.

Place the desiccator inside the incubator under controlled temperature and darkness during all larval development and emergence of the adults. It is recommended to transfer the samples to another desiccator at least every 3 d to avoid mold growth on the walls.

**Note:** When manipulating the desiccator, avoid sudden movements because eggs and initial larval stages are the most sensitive stages to movement and manipulations. The temperature should be continuously monitored using a minimum/maximum thermometer kept in the incubator.

When the feeding period of larvae finishes, it is necessary to reduce the RH to ~70 ± 10% to simulate natural conditions. To do this, replace the water from the Petri dish (which is inside the desiccator) with a saturated solution of NaCl. The salt solution must be maintained until adult emergence.

**Note:** Replace the salt solution every 4–5 days to avoid mold growth and refill the solution if it evaporates.

Upon emergence, mark the adult bees near the tip of their thorax using nontoxic water-soluble paint. Make sure to prevent the paint from sticking to the wings. This procedure allows the bees to be monitored at different ages within the groups.

Adult bees can be kept in plastic pots or Petri dishes with a sucrose solution feed *ad libitum*. Each pot or Petri dish must receive the bees from the same microplate (i.e., from the same treatment and colony origin), and they should be maintained under controlled conditions of temperature and RH (in darkness) for the subsequent sublethal assessments.

**Note:** Stingless bees are eusocial insects, they must be kept in groups of adults to ensure greater survival [10].

### Mortality assessment and observations

**Fig. 4:**
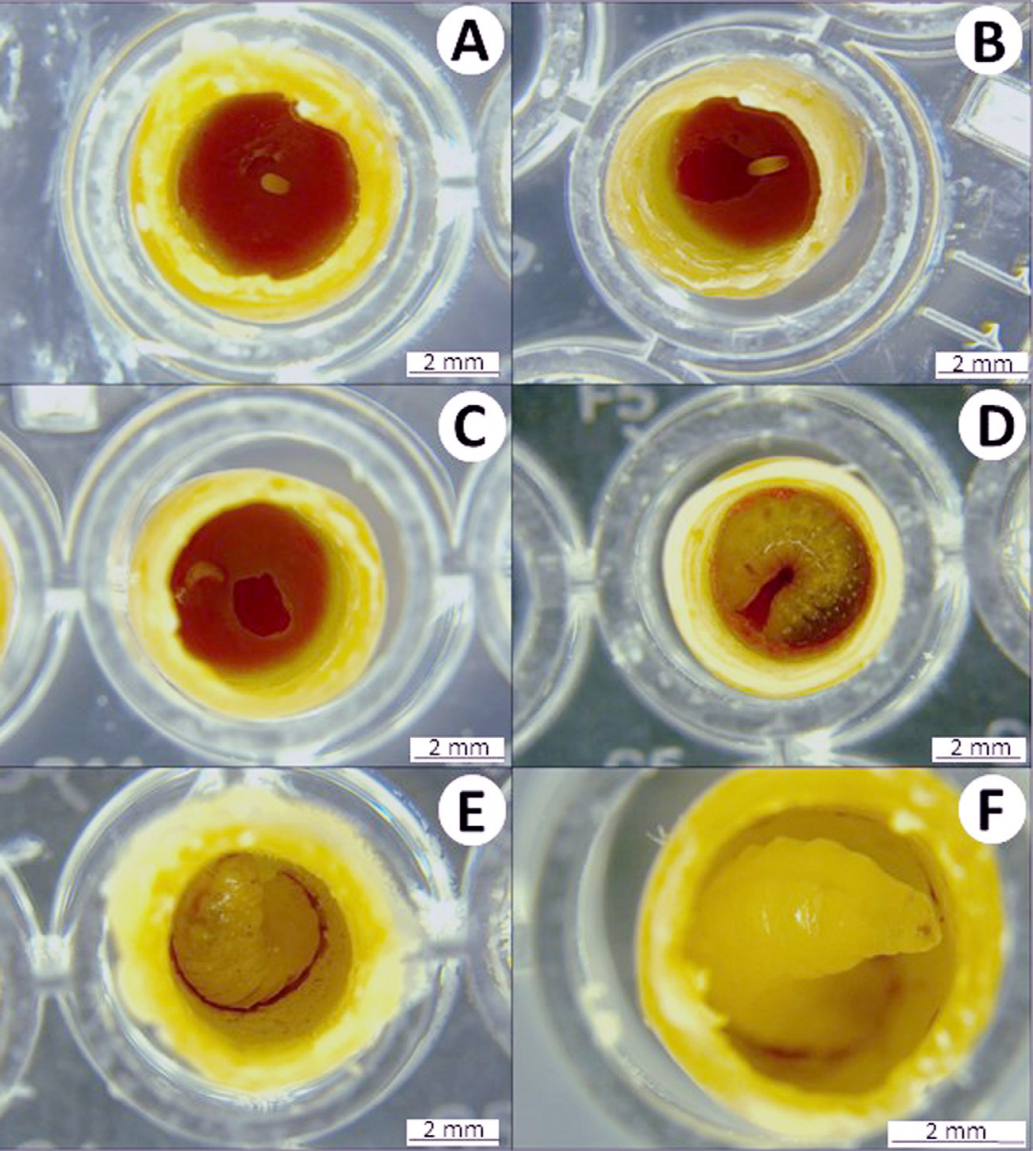
Egg and larval development stages of *Partamona helleri* workers. (A) Egg on larval food post grafting; (B) larva on the first day after egg hatching; (C) larva on third day after hatching; (D) larva at the end of feeding period on the ninth day after hatching; (E) defecating larva on 12th day after hatching, and (F) larva on the 15th day post hatching.

**Note:** Do not touch the larvae while assessing mortality to avoid injures. Return the individuals to their appropriate desiccator as soon as completing the mortality assessment. If the studied species has very small larvae, use a stereo microscope to determine larvae mortality by spiracle movements and larvae body contractions.

**Fig. 5:**
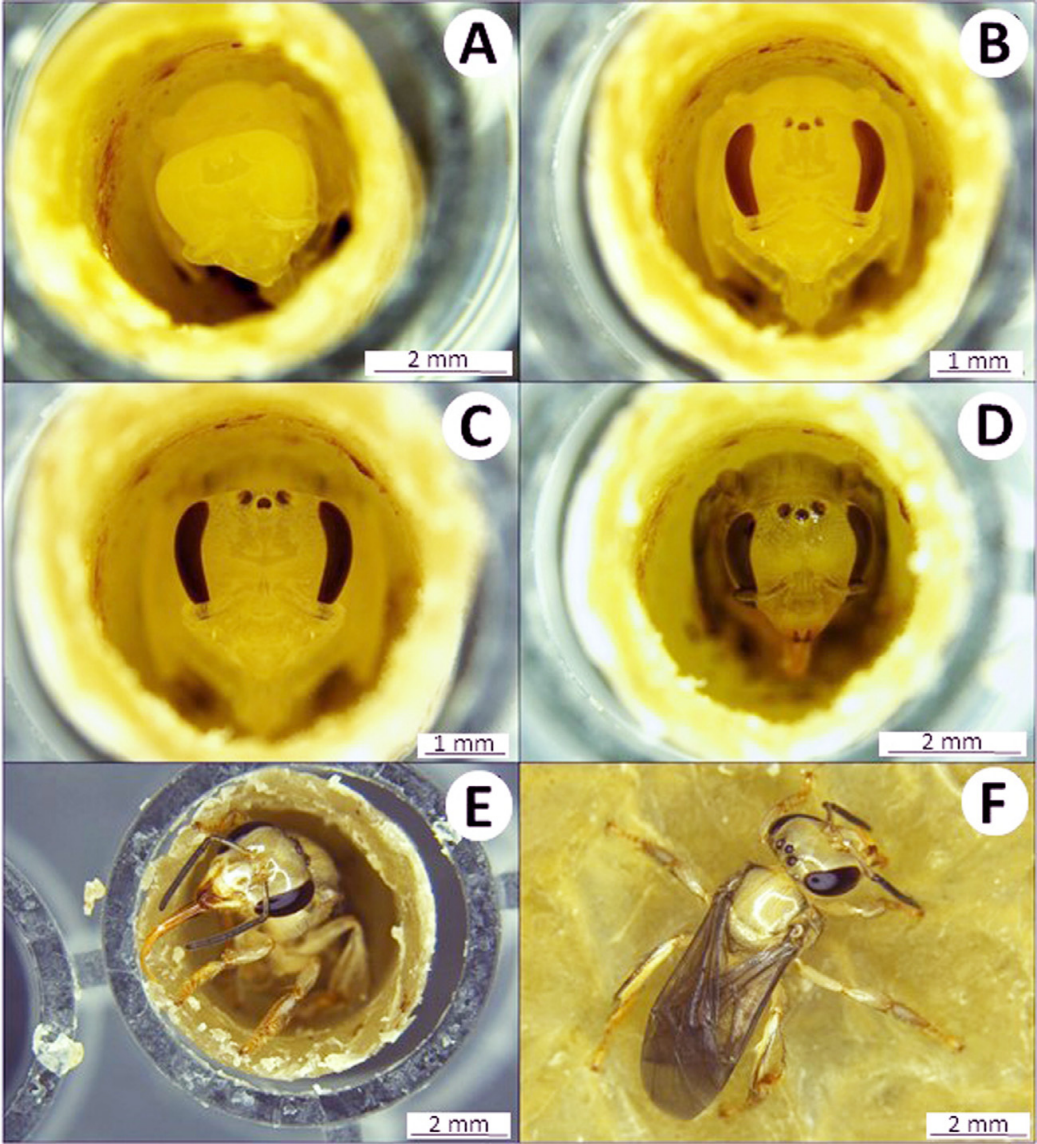
Pupae development of *Partamona helleri* workers. (A) Pupae on the 24th day after egg hatching with the white eye; (B) pupae on the 33th day after hatching with pink eye; (C) pupae on the 40th day after hatching with brown eye; (D) pupae on the 47th day after hatching with black eye and body hair; (E) and (F) newly-emerged adult on the 49th day after hatching.

**Fig. 6:**
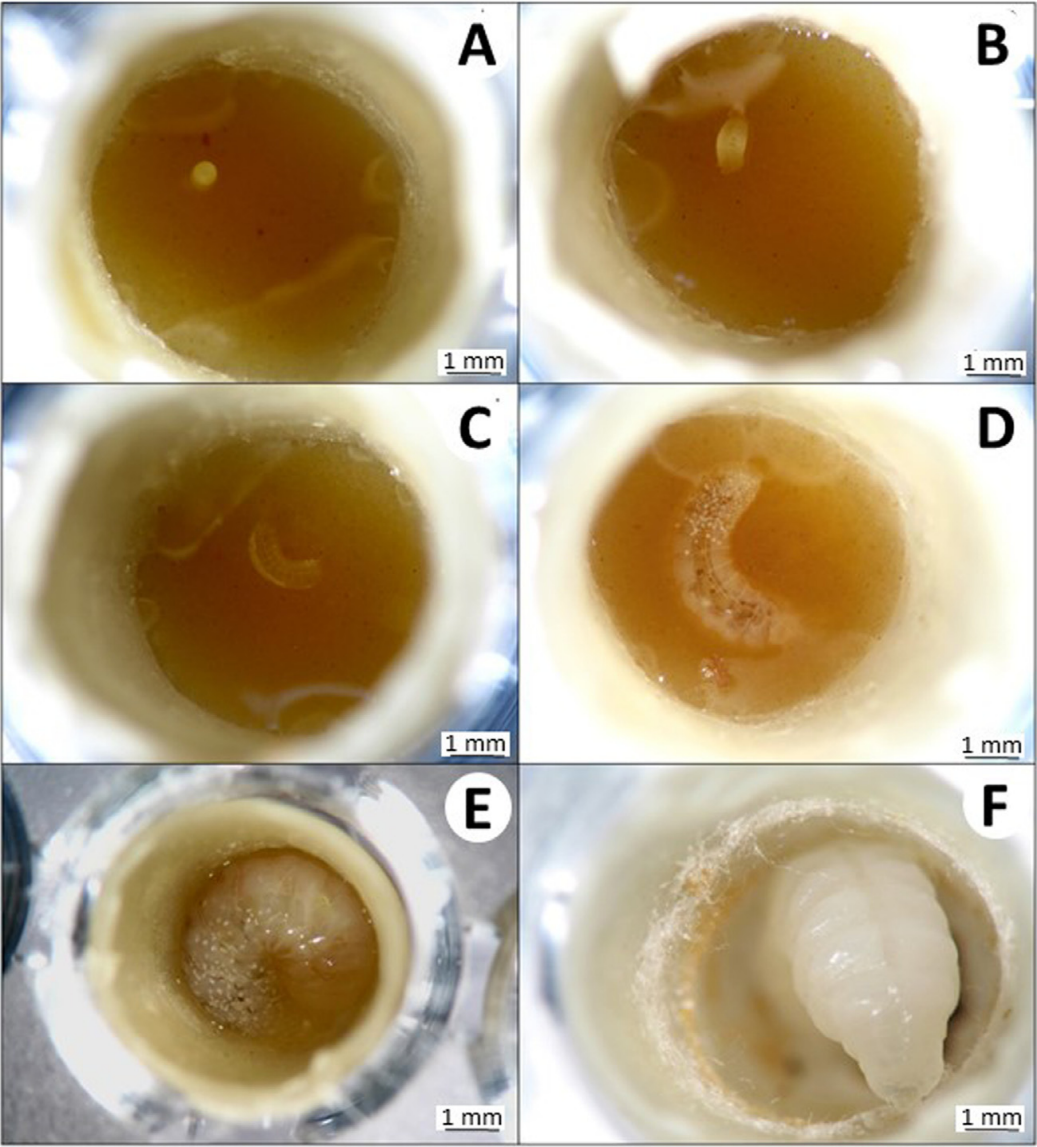
Egg and larval development stages of *Trigona spinipes* workers. (A) Egg on larval food post grafting; (B) larva on the first day after egg hatching; (C) larva on the second day after hatching; (D) larva on the sixth day after hatching; (E) larva on the ninth day after hatching, and (F) larva on the 15th day after hatching.

**Fig. 7:**
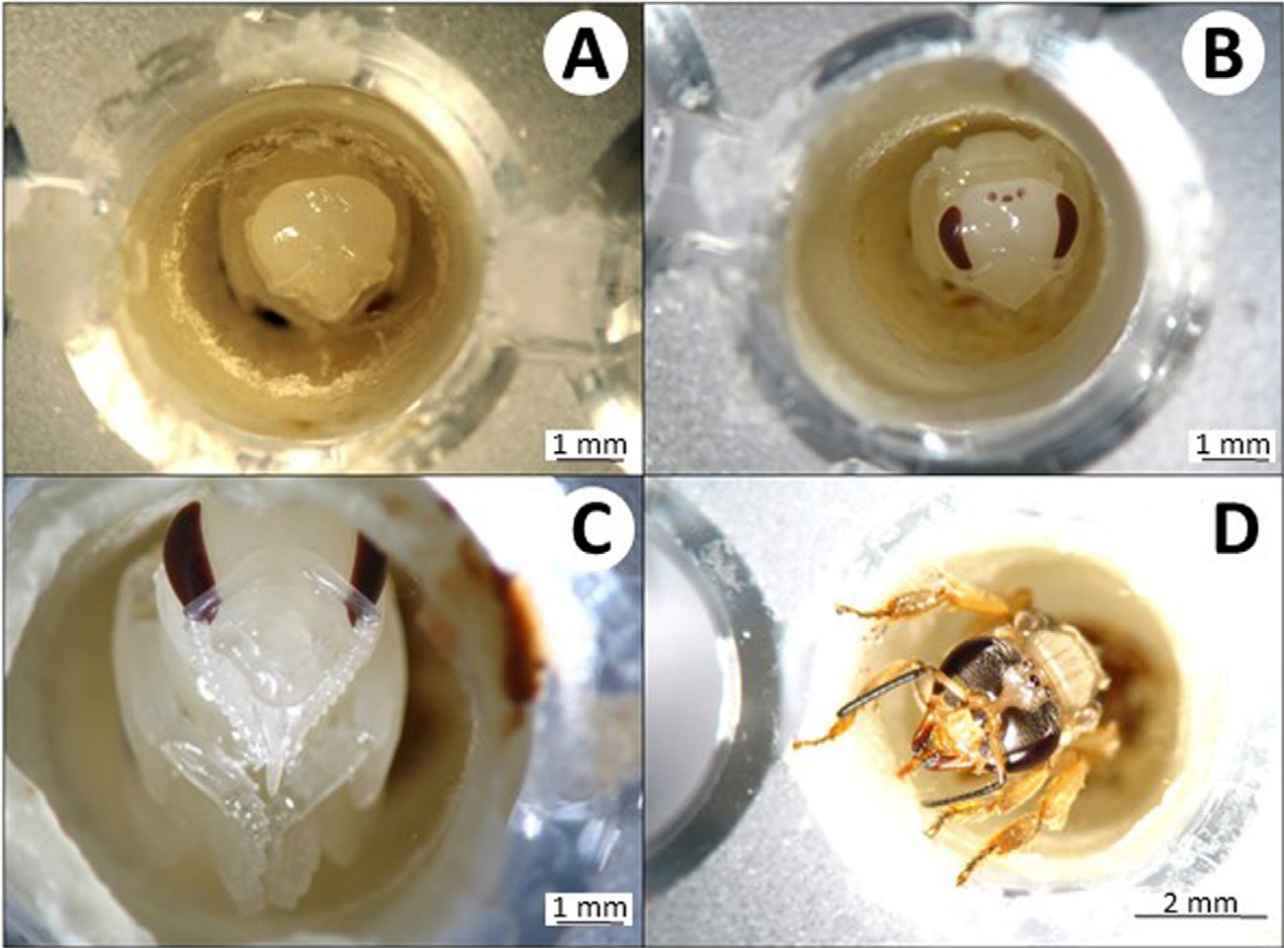
Pupae development of *Trigona spinipes* workers. (A) Pupae on the 18th day after with the white eye after hatching; (B) pupae on the 26th day with brown eye after hatching; (C) pupae on the 28th day after hatching with the brown eye, and (D) newly-emerged adult on the 35th day post hatching.

Larval survival and its development should be monitored daily (Figs. 4–7). Larvae will be incubated in darkness to simulate hive conditions. However, larvae can be exposed to laboratory lighting for 30 min each day during survival monitoring. Larvae that are injured by handling during data collection must be censored from the analysis.

**Fig. 8:**
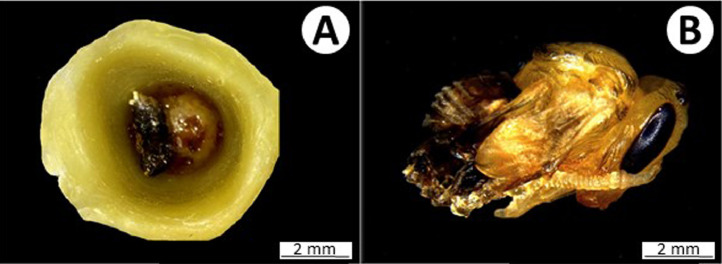
Morphological abnormalities in *Partamona helleri* queen. (A) A dead larva with lack of turgidity and presence of black spots. (B) Pupa with malformed appendages.

Remove dead individuals to avoid undesired microbial growth (Fig. 8 (A)). Dead individuals can be recognized by the following symptoms: absence of movement, deflated/flaccid body, lack of turgidity, and the presence of black spots on the body.

Sublethal effects, such as malformation of appendages (Fig. 8 (B)), as well as changes in developmental time, can be assessed during pupation. Body mass can be determined during the pupae or adult phase. Bees should be carefully removed from the artificial cell for weighing. However, this procedure is very risky for the pupae because they are more susceptible to handling than newly emerged adults. In all assessments, avoid sudden movements.

In general, only the mortality of workers is included in the analysis, but males and queens can be included depending on the study. The sex of individuals can be determined at the dark-eyed pupal stage using a stereomicroscope.

**Note:** Queens are recognized by the presence of 10 antennal flagellomeres, the absence of corbicula at their hind tibia, and small compound eyes compared with those of workers. Males are recognized by the presence of 11 antennal flagellomeres, gonopods, different external morphology of their abdomen compared with that of females and the absence of corbicula [11].

## Methods validation

We performed a meta-analysis based on results from previous published works that used the protocols for ecotoxicological bioassays with the stingless bees considered here. For acute adult exposure, we found five studies with 29 independent controlled bioassays with binary outcomes (mortality) and calculated the risk ratio (RR) [12]. For chronic larva exposure, we found six studies with 10 independent controlled bioassays. Furthermore, we measured the effects of chronic larval exposure on survival (time to event data) and calculated hazard ratios (HR) using the method described by Tierney et al. [13]. We measured heterogeneity and, when necessary, subgroup analyses were performed to explain the statistical heterogeneity. The results were validated according to the comparison of the survival rate or mortality rate between bees treated with agrochemical and untreated bees (control) for both acute and chronic exposures. Thus, two meta-analyses were undertaken separately on acute and chronic exposures of adults and larvae, respectively. For both types of bioassays (i.e., chronic and acute), we used mixed-effect models (heterogeneous subgroups). We evaluated the occurrence of the publication bias by funnel plot and rank correlation test [14]. The meta-analyses were performed using the meta-package [15] in the R software [16].

The protocols described above were adapted for each species regarding temperature, humidity, light, and amount of larval food (Tables 1 and 2). However, the performance scheme of toxicological tests did not change, which means that these protocols can be used for different species of stingless bees. In addition, the survival rate obtained for both acute and chronic exposure was ≥80% on untreated bees with agrochemicals in all studies mentioned here, supporting the use of these protocols for suitable toxicological assessment.

Acute adult exposure was performed via oral or contact, and both methods proved to be successful for toxicological tests while maintaining high survival of adult bees in the controls (=100%) until the end of the tests, which used *M. quadrifasciata, Friesella schrottkyi, P. helleri*, and *Scaptotrigona xanthotricha* (Table 1).

Chronic larval exposure was performed by the *in vitro* rearing method. These tests enabled the evaluation of sublethal and lethal effects of agrochemicals during the development of *M. quadrifascita* [5,^7^,17], *P. helleri* [6,18], and *T. spinipes* [4] under laboratory conditions, which were suitable for toxicological studies and exhibited an average survival rate of 85% (Table 2). The results showed a high hatching rate of eggs in an uncontaminated diet and successful larval development followed by healthy adult emergence. In addition, adults did not exhibit morphological deformations compared to larvae fed on a contaminated diet. It was eggs instead of larvae (≥90% hatching rate), which were transferred to the artificial wax cells, that allowed the assessments of each individual and its exposure to the contaminated diet from the first larval instar. Egg grafting is a critical step for chronic exposure, which requires training and practice before the beginning of rearing because the eggs are very susceptible to injuries, which may compromise hatching.

The volume of the larval diet varied depending on the stingless bee species. Therefore, we used an amount of larval food adequate for each species, which was previously described in other studies. For example, each worker of *M. quadrifasciata, P. helleri*, and *T. spinipes* received, respectively, 140, 40, and 36 µL of food and each queen of *P. hellleri* received 80 µL of food. These amounts of food were sufficient for the full development of the individuals. The developmental stages of *P. helleri* are represented in Figs. 4 and 5 and *T. spinipes* in Figs. 6 and 7. A dead larva and a deformed pupa are depicted in Fig. 8.

**Fig. 9:**
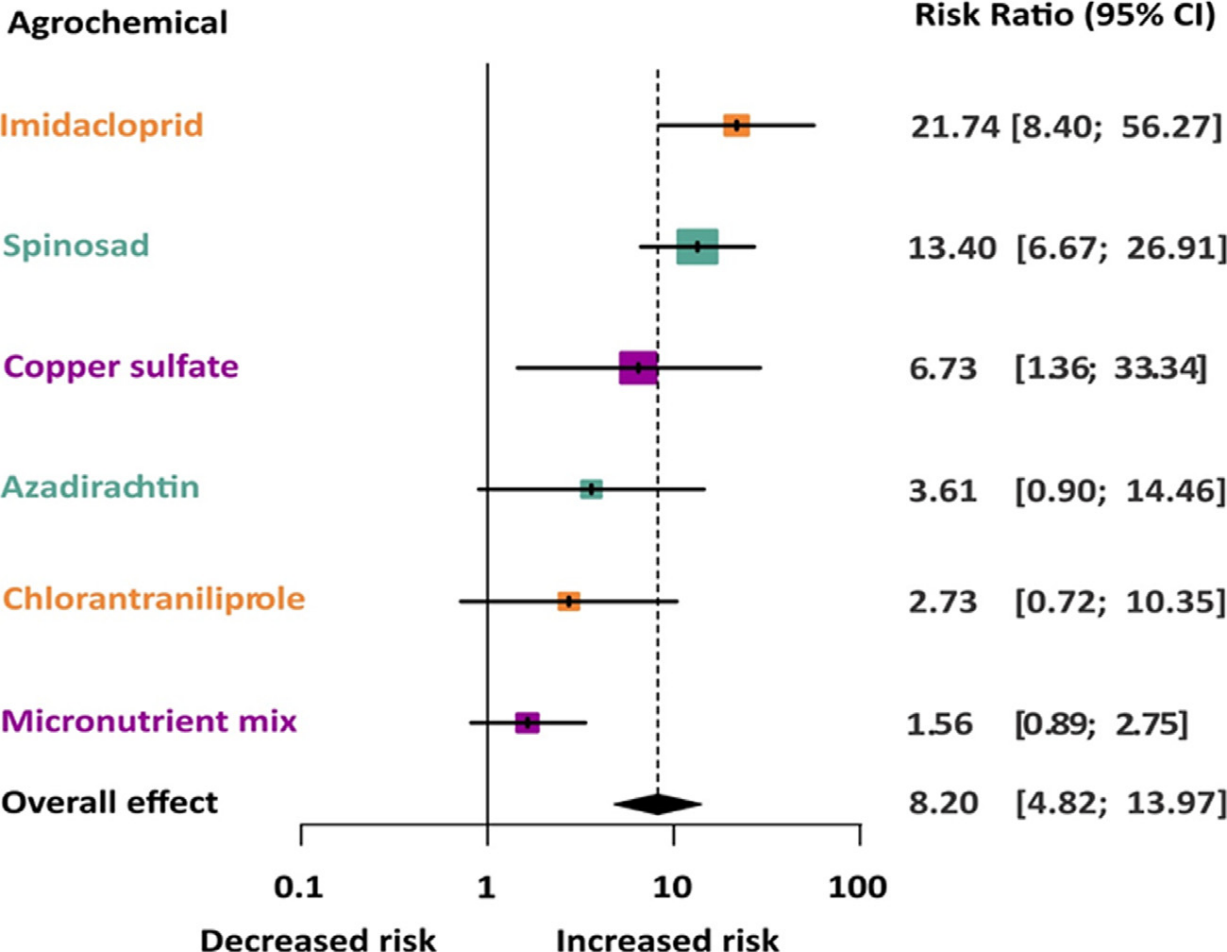
Estimated effects (mixed-effect model) of acute adult exposure to agrochemicals on the mortality of stingless bees according to the type of agrochemical. For each type of agrochemical, it is showed the estimated risk ratio (RR) and its 95% confidence interval (CI). The area of the gray square centered on the risk ratio is the inverse of the variance (larger squares indicate studies with more precise results, i.e. small variances). On the left side of the forest plot, it is showed the RR and its 95% confidence interval. The black diamond shape indicates the summary RR (overall effect). Different color indicates the type of agrochemical: insecticides (orange), bioinsecticides (green) and leaf fertilizers (purple).

The meta-analyses of acute adult exposure showed low survival of treated bees when compared with that of the control. The risk ratio (RR) was significantly higher than expected by the null hypothesis (RR = 8.2, 95% CI = 4.82 - 13.97, *z* = 7.75, *p* < 0.0001). To explain the high heterogeneity (Cochran's Q statistic = 84.34, df = 22, *p* < 0.001, I2 = 73.9%), we also included the type of agrochemical as a moderator in the model, which explained the heterogeneity among groups (Cochran's Q statistic = 34,21 df = 5, *p* < 0.0001). Therefore, there were significant survival decreases because of acute adult exposure to agrochemicals that varied with the type of compound (Fig. 9). Imidacloprid (RR = 21.74), spinosad (RR = 13.40), and copper sulfate (RR = 6.73) exhibited the greatest risk for the survival of bees as compared with that of azadirachtin, micronutrient mix, and chlorantraniliprole (non-significant RR). Our protocols were also successfully used to evaluate sublethal effects, which included behavioral, physiological, morphological, and molecular assessments. Responses to sublethal concentrations varied according to the type of agrochemical and species of stingless bees [19], [20], [21], [22], [23].

**Fig. 10:**
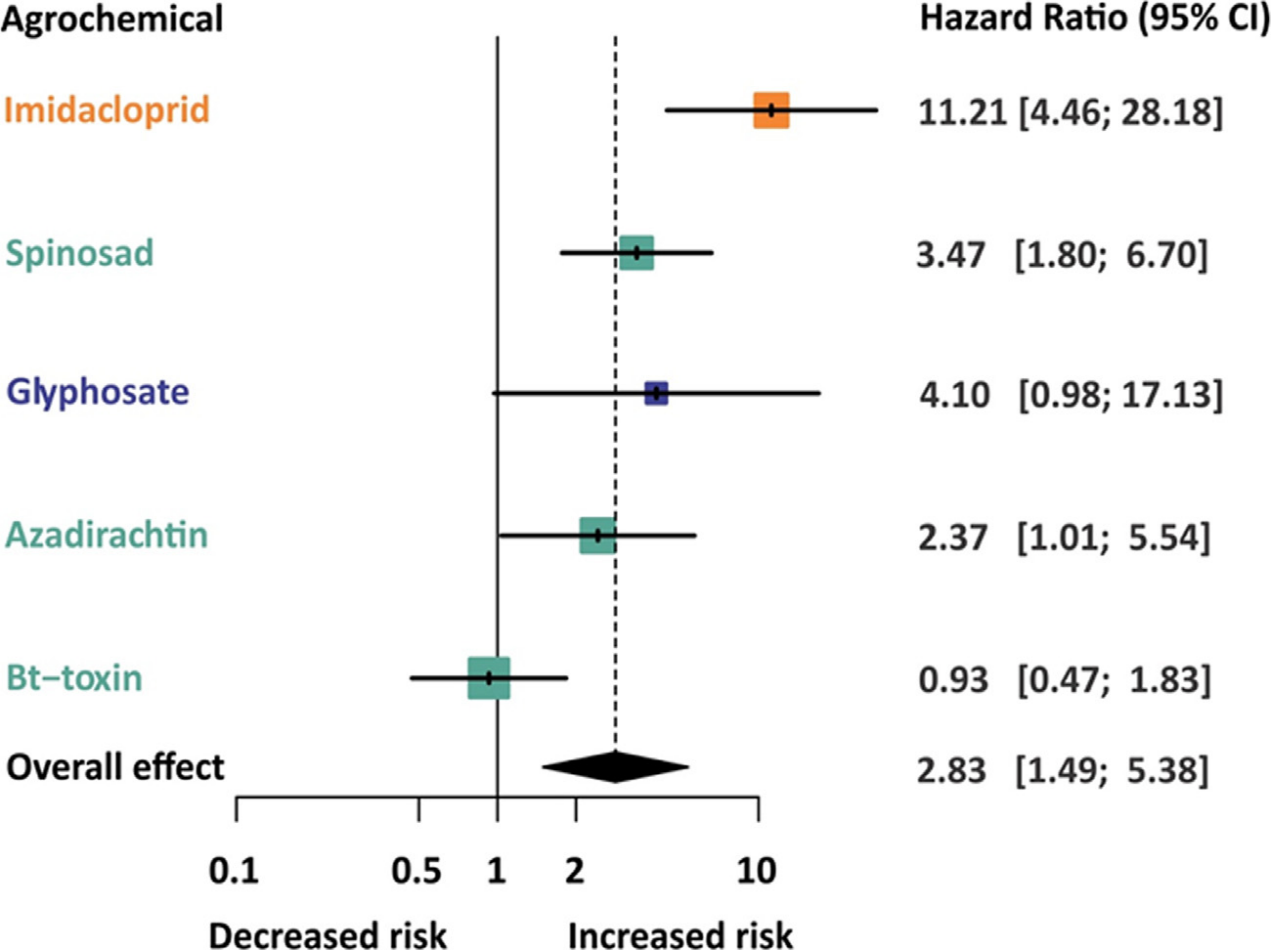
Estimated effects (mixed-effect model) of chronic exposure to agrochemicals on the survival of the stingless bee according to the compound. For each type of agrochemical, the estimated hazard ratio (HR) and its 95% confidence interval (CI). The area of the gray square centered on the estimated hazard ratio is the inverse of the variance (larger squares indicate studies with more precise results, i.e. smaller variances). On the left side of the forest plot, HR and its 95% confidence interval are depicted. The blue diamond shape indicates the summary of HR (overall effect). Different color indicates the type of agrochemical: insecticides (orange), bioinsecticides (green) and herbicide (blue).

The results of the meta-analysis considering the chronic larval exposure resulted in an estimated model for the HR that was significantly higher than expected by the null hypothesis (HR = 2.83, 95% CI = 1.493–5.376, *z* = 3.19, *p* = 0.0014). As there was significant heterogeneity (Cochran's Q statistic = 48.14, df = 9, *p* < 0.001, I2 = 81.3%), we performed subgroup analysis with the agrochemical type as moderator. The subgroup analysis showed that the agrochemical type explained the statistical heterogeneity among groups (Cochran's Q statistic = 19.63, df = 4, *p* = 0.0006). Imidacloprid and spinosad exhibited a greater risk to survival rates (HR = 11.21 and 3.47, respectively). In contrast, azadirachtin (HR = 2.37) and Bt-toxin (HR = 0.93) did not exhibit risk and glyphosate (HR= 4.1) exhibited a survival risk but it was not significant. The results for chronic larval exposure were similar to those of acute adult exposure. Survival decreased because of the type of agrochemical, as shown in Fig. 10.

Finally, our results also showed that the published papers using our rearing protocol did not have a publication bias for either acute exposure (*z* = 0.35, *p* = 0.73) or chronic exposure (*z* = 0.72, *p* = 0.47). This indicates the suitability of the protocols, which can be applied to other Meliponini species.

## Additional information

Agrochemical formulations considered in the analyses and commonly used in Brazil at their respective label rates [24]:

- Insecticides:
- Chlorantraniliprole (Premio^Ⓡ^; suspension concentrate at 200 g a.i. L^−1^ (active ingredient/L) DuPont, Barueri, SP, Brazil), which acts on the ryanodine receptor via stimulation of the release of calcium in muscle cells, causing individual paralysis and death [25].
- Imidacloprid (Evidence^Ⓡ^; water dispersible granules at 700 g a.i. kg^−1^, Bayer CropScience, São Paulo, SP, Brazil), which is an agonist of nicotinic acetylcholine receptors (nAChRs) in the nervous system [26].
- Bioinsecticides:
- Spinosad (Tracer^Ⓡ^; suspension concentrate at 480 g a.i. L^−1^, Dow AgroSciences, Santo Amaro, SP, Brazil), which acts as an agonist of nAChRs and γ-aminobutyric acid (GABA) receptors, leading to hyperexcitation [27].
- Azadirachtin (Azamax^Ⓡ^; emulsifiable concentrate at 12 g a.i. L^−1^, DVA Agro Brazil, Campinas, SP, Brazil, and Cursor^Ⓡ^; emulsifiable concentrate at 10 g a.i. kg^−1^; BIO CARB, Curitiba, PR, Brazil), which is a growth regulator and anti-feeding agent [28].
- Cry1Ac (Bt-toxic 1.8 µg toxin larva^-1^, Biochemistry Department of Case Western Reserve University, Cleveland, OH, USA). Their primary action is to lyse midgut epithelial cells, compromising the peritophic matrix and gut membrane that may lead to septicemia [29];
- Leaf fertilizers:
- Copper sulfate (Sulfato de Cobre Penta 24^Ⓡ^; a salt formulation containing 240 g kg^−1^ Cu and 110 g kg^−1^ S; Multitécnica Industrial, Sete Lagoas, MG, Brazil). The heavy metals present in its composition can inactivate many enzymes by replacing essential metal ions in biomolecules, resulting in their inhibition or function loss [30].
- Micronutrient mix (Arrank L^Ⓡ^; homogeneous suspension containing (w/v) 4.00% S, 0.50% B, 0.60% Cu, 3.00% Mn, 0.06% Mo, and 5.00% Zn, corresponding to 50.80, 6.35, 7.62, 38.10, 0.76, and 63.50 g L^−1^, respectively, in the formulation; Quimifol, São Paulo, SP, Brazil), Its toxicity is caused by the presence of heavy metals that act in the inhibition of vital enzymes [30].
- Herbicide:
- Glyphosate (Roundup Original Di^Ⓡ^; soluble concentrates at 445 g a.i. L^−1^ of Di-ammonium salt of N-(phosphonomethyl) glycine, 370 g a.i. L^−1^ of the acid equivalent of N-(phosphonomethyl) glycine; Monsanto do Brazil, São José dos Campos, SP, Brazil). It disrupts the shikimic acid pathway that is vital for protein synthesis only found in microorganisms and plant growth. Glyphosate is absorbed across the leaves and stems and is translocated throughout the plant [31].
